# Population Frequency of Undiagnosed Fabry Disease in the General Population

**DOI:** 10.1016/j.ekir.2023.04.009

**Published:** 2023-04-17

**Authors:** Amalia Kermond-Marino, Annie Weng, Selina Kai Xi Zhang, Zac Tran, Mary Huang, Judy Savige

**Affiliations:** 1 Department of Medicine, Melbourne Health and Northern Health, The University of Melbourne Victoria, Australia

## Abstract

**Introduction:**

Fabry disease is an X-linked disorder that results from pathogenic *GLA* variants and can now be treated. Most studies of its population frequency have examined only males or attendees at kidney failure or cardiac clinics. This study determined the prevalence of undiagnosed Fabry disease from predicted pathogenic *GLA* variants in the general population.

**Methods:**

The Genome Aggregation Database (gnomAD) was examined for predicted pathogenic *GLA* variants based on variant rarity (≤5), and transcript effect in 4 computational tools (CADD >20, PP2 >0.95, SIFT <0.05, Mutation Taster – Disease-causing) and amino acid conservation in vertebrates in a Clustal.

**Results:**

Predicted pathogenic variants in *GLA* occurred in 1 in 3225 of the gnomAD population and 1 in 3478 of its control subset. Predicted pathogenic variants were more common in women than expected (3.1:1), which is consistent with men being excluded from gnomAD because of Fabry complications. Predicted pathogenic variants were not found in members of this cohort with South Asian, Ashkenazim, or Finnish ancestries. Variants identified as pathogenic in the Fabry database were found in 1 in 2651 individuals of the gnomAD database and pathogenic variants from ClinVar in 1 in 4420.

**Discussion:**

The population frequency of 1 in 3225 for undiagnosed men and women with Fabry disease still represents an underestimate because our pathogenicity criteria were rigorous, the cohort did not include already-diagnosed individuals, and whole exome sequencing does not detect intronic variants and large deletions. This study confirms that Fabry disease is more common than previously recognized and still underdiagnosed especially in women.

## Graphical abstract

**Figure:**
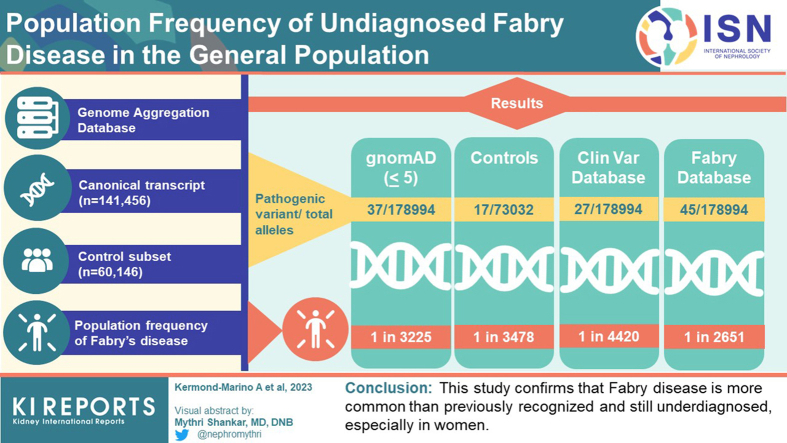

Fabry disease (MIM301500) is an X-linked disorder characterized by progressive kidney failure, cardiac disease and stroke, peripheral neuropathy, and angiokeratoma.^1^ It is caused by pathogenic variants in the *GLA* gene that affect the lysosomal enzyme α-galactosidase A activity, and result in the accumulation of globotriaosylceramide (Gb3) and related glycosphingolipids in the blood vessels of affected tissues.^1^

Males with the “classical” or renal variant of Fabry disease have little or no functional enzyme activity, and the accumulation of microvascular endothelial glycosphingolipid is associated with the onset of clinical features during childhood or adolescence. Affected males initially develop proteinuria and most have end-stage kidney failure by the age of 50 years.^2^ Most of the affected individuals in dialysis units have classical disease.

There is an “atypical” form of Fabry disease with residual enzyme activity and a later onset of cardiac features sometimes accompanied by kidney failure.^3^ Males typically present after the age of 50 with left ventricular hypertrophy, cardiomyopathy, arrhythmias, or cryptogenic stroke. Atypical Fabry disease may be much more common than the classical form.^4^

Because the inheritance of Fabry disease is X-linked, twice as many women are affected as men. Some affected women also develop kidney failure and heart disease,^5^ and even where they have no clinical features, half their sons and daughters inherit the pathogenic variant.

Treatment for Fabry disease is now available in the form of enzyme replacement and a chaperone that delay kidney failure onset and cardiac abnormalities.^6^^,^^7^ Early treatment prevents irreversible organ damage.

Recognition of Fabry disease is important to predict organ involvement, to identify and treat other affected family members, and because of the availability of treatment. Knowing the true population frequency of Fabry disease indicates to clinicians how often it is likely to affect their patients.

Many studies have estimated the frequency of Fabry disease using newborn screening and biochemical testing; renal biopsies; rare disease registries; as well as genetic testing in renal, cardiac, and stroke clinics. Population frequencies have varied from 1 in 1222 to 1 in 9372 using a fluorometric enzyme assay or mass spectrometry in Japanese and American males.^8^^,^^9^ One study where enzyme tests were confirmed with DNA sequencing in Italian newborn boys found 1 in 7879 had a pathogenic variant consistent with later onset disease.^10^ One in 6000 renal biopsies had features consistent with Fabry disease.^11^ One in 400 males attending a hemodialysis or kidney transplant clinic had Fabry disease,^2^ and 1 in 100 in a cardiac clinic with left ventricular hypertrophy or hypertrophic cardiomyopathy and 1 in 800 with stroke were affected. However, biochemical assays are inaccurate and difficult to interpret in females,^10^ kidney biopsies may not have distinctive features in late disease, and identifying affected individuals in renal and cardiac clinics is too late to start treatment.

The diagnosis of Fabry disease is made definitively with genetic testing and the demonstration of a pathogenic *GLA* variant. Although genetic testing of an unselected cohort is the most accurate method of determining the population frequency of Fabry disease, making a conclusive diagnosis remains difficult. Genetic variants are typically different in each family, there are no mutational “hotspots” (Supplementary Figure S1), and most are missense changes, where it is difficult to identify pathogenicity with certainty. Therefore, expert panels have revised the status of some variants that were previously considered “pathogenic” to “benign”^12^.

This study examined the gnomAD website for pathogenic variants consistent with the diagnosis of Fabry disease.^13^ This strategy has been used previously for rare variants in Alport syndrome, Gitelman syndrome, autosomal dominant polycystic kidney disease, mucopolysaccharidoses, and Menke disease.^14^, ^15^, ^16^, ^17^, ^18^ In at least autosomal dominant Alport syndrome and Gitelman syndrome, these analyses confirmed population frequencies that had been obtained using alternative means such as renal histology in transplant donors^19^ and a hospital cohort with hypokalemia respectively.^15^ We defined pathogenicity based on American College of Medical Genetics and Genomics criteria,^20^ evaluated our strategy, and compared it with examining variants assessed as pathogenic in ClinVar or the Fabry database. We also examined why these individuals were undetected (female sex, atypical form) and whether they were more common in people of any ancestry.

## Methods

### Database

Genetic variants (GRCh37/hg19) from gnomAD (v2.1.1, www.gnomAD.broadinstitute.org) canonical transcript (*n* = 141,456) and its control subset (v2.1.1, controls *n* = 60,146) were used to estimate the population frequency of Fabry disease (downloaded 10 June 2022). gnomAD comprises aggregated information from 125,748 whole exome sequencing and 15,708 whole genome sequencing outcomes of unrelated individuals with adult-onset diseases such as diabetes, cardiac or neuropsychiatric disease, after excluding any with a severe pediatric disease and their family members. It included equal numbers of males and females. The sex, age range, and ancestries, but no clinical data, were available. The control subset represented data from individuals recruited as controls for these studies who were examined separately to exclude bias from participants cardiac involvement due to Fabry disease.

The scores available for gnomAD indicated that variants in *GLA* were constrained for loss-of-function changes, with a pLi = 1, observed/expected = 0 (95% confidence interval of 0−0.18) but were not constrained for missense changes with a Z = 1.88, o/e = 0.58 (0.49–0.69).

All gnomAD participants had provided written informed consent for the use of their anonymized data at the time of recruitment and institutional review board approval was not required for this project.

### Filtering Steps

Pathogenic variants were presumed to be rare and to affect gene coding regions. Variants located in the 5′ and 3′ UTR, and in the intronic and noncoding transcripts were excluded. Variants that resulted in synonymous changes or affected splice regions other than in canonical positions were also excluded. Finally, variants that were homozygous or found more than 5 times in gnomAD were excluded. This number was chosen based on the disease prevalence, the likely contribution of an individual variant, and the high disease penetrance.

Variants were then filtered according to their effect on the canonical transcript (ENST00000218516.3) using the following steps. The term “predicted pathogenic” was used for variants that passed all filtering criteria, to differentiate them from the “pathogenic” and “likely pathogenic” variants derived from the American College of Medical Genetics and Genomics/Association for Molecular Pathology classification^20^ in individuals with clinical features of disease.

Variants that resulted in a protein-truncating change, frameshift, or canonical splice site change were designated predicted pathogenic because loss-of-function is a known disease mechanism for *GLA*-associated disease.

Missense variants were then filtered with various computational tools as follows: CADD (> 20); Polyphen-2 (PP2, score ≥0.95, www.genetics.bwh.harvard.edu/pph2/); SIFT (Sorting Intolerant from Tolerant, Deleterious, www.sift.bii.a-star.edu.sg/); and Mutation Taster, (MT, Disease-causing, and conservation in vertebrates (including birds) (Clustal, Supplementary Figure S2) (www.asia.ensembl.org/). Amino acid substitutions that resulted in a similar amino acid type (same color in the Clustal) were considered conserved. Variants that fulfilled all these criteria were designated “predicted pathogenic.”

### Evaluation of Strategy

The filtering strategy was evaluated using variants from the Fabry database as follows: (i) associated with the classical disease, or (ii) milder/later onset disease; (iii) that were considered a variant of uncertain significance (VUS) in the database, or (iv) that were found in more than 10 individuals in the gnomAD database and therefore assumed to be benign. These values were then used to calculate the sensitivity; specificity; as well as positive and negative predictive values for severe, late onset, and VUS compared with predicted pathogenic variants from nonpathogenic variants in the Fabry database (*n* = 4), common in gnomAD or benign in ClinVar (*n* = 15) (Table 1). All calculations assumed that only 1 pathogenic variant was found in each person.

**Table 1:** Accuracy of filtering strategy for pathogenic variants in *GLA*

Assessment characteristic	“Pathogenic” variants in Fabry db			? VUS in Fabry db	“Benign” variants		
	All Fabry (*N* = 67)	Classical disease (*n* = 30)	Later onset disease (*n* = 37)	(*n* = 36)	Common in gnomAD or Benign in Clin Var (*n* = 15)	Benign in Fabry db (*n* = 4)	Total negative controls (*N* = 19)
Sensitivity	46/67 (69%)	25 (83%)	21 (57%)	11 (31%)	0 (0%)	0 (0%)	0 (0%)
Specificity	19/19 (100%)						
PPV	46/46 (100%)						
NPV	19/40 (48%)						

Of the 30 variants associated with classic disease, 25 (83%) were predicted pathogenic by our strategy (Supplementary Table S1). Of the 37 variants associated with atypical or later onset disease, 21 (57%) were predicted pathogenic. Of the 36 variants assessed as a VUS in the Fabry database, 11 (31%) were predicted pathogenic (Supplementary Table S2).

Of the 24 normal variants, none was predicted pathogenic with our strategy (Supplementary Table S3).

This meant that our approach had a sensitivity of 83% for classic disease, 57% for atypical disease, and 31% for a VUS (Table 1). Overall, for the classical and atypical forms of Fabry disease the sensitivity was 46 of 67 (69%) but with a high positive predictive value (46/46, 100%) and a negative predictive value of 48% (19/40).

### Calculation of the Population Frequency in gnomAD

The number of pathogenic or predicted pathogenic variants was then considered for the number of individuals in gnomAD, considering that women have 2 X chromosomes and men have only 1.

The population frequency of our predicted pathogenic variants was also examined in the control subset.

### Population Frequencies of Variants of Different Ancestries in gnomAD

The population frequencies of the predicted pathogenic variants were examined in people from each of the 8 ancestries reported in gnomAD.

### Features That Mitigated Phenotype for Predicted Pathogenic Variants in gnomAD

Finally, the cohort with predicted pathogenic *GLA* variants was examined to determine how often variants were found in women or associated with atypical or mild disease that might explain why the diagnosis had not already been made clinically.

### Population Frequencies Using Different Databases and Criteria

Different population frequencies were then compared; for the predicted pathogenic variants using this strategy; variants that were assessed previously as pathogenic on the Fabry disease website (http://fabry-database.org/); or where variants were predicted pathogenic in ClinVar (https://www.ncbi.nlm.nih.gov/clinvar/); and from the Fabry expert panel.^12^

## Results

### *GLA* Variants in gnomAD

gnomAD included 1 null and 99 missense variants in a mean of 178,994 alleles that comprised equal numbers of males and females and corresponded to 119,329 individuals.

### Population Frequency of Predicted Pathogenic Variants in gnomAD

There were 20 variants in 37 individuals from gnomAD that were assessed as predicted pathogenic variants (Tables 2 and 3). This corresponded to a population frequency of 37 of 119,329 or 1 in 3225.

**Table 2:** Population frequency of pathogenic variants in gnomAD using our filtering strategy and controls, and ClinVar or the Fabry database assessment

Allele and population frequencies	gnomAD (≤5)	Controls	Clin Var – P/LP or VUS/LP	Fabry db
	20 variants in 37 people	11 variants in 14 people	13 variants in 27 people	28 variants in 45 people
Pathogenic variant/total alleles	37/178,994	14/73,032	27/178,994	45/178,994
Population frequency	39/119,329	14/48,688	27/119,329	45/119,329
Population frequency	One in 3225	One in 3478	One in 4420	One in 2651

These results suggest that the population frequency of undiagnosed Fabry disease in a normal adult population is about 1 in 3225 or 1 in 3478 in a control group.

**Table 3:** Predicted Pathogenic *GLA* variants in the gnomAD normal database from variants considered pathogenic

Variant	Phenotype	Number, sex	Age	Ancestry	Controls	ClinVar	Reference in Fabry db
p.Gly395Ala		2F	60–65, 75–80	2 Euro	2F, 60–65, 75–80	VUS	Bono 2011^21^
p.Pro 343Leu		1M	1M, 70–75	1 Euro	1M, 70–75	Not reported	Mills 2005^22^
p.Pro343Ser		1M	1M, 40–45	1 Euro	0	Not reported	Not reported
p.Met290Ile		1F	1F, 60–65	1 Other	1F, 60–65	Conflicting (P/LP/VUS)	Siekierska 2012^23^
p.Ala285Thr		1F	1F, 60–65	1 Other	0	Not reported	Not reported
p.Asp264Asn		1F	Not known	1 Euro	1F	VUS	Lukas 2013^24^
p.Lys240ArgfsTer29		1F	Not known	1 Euro	0	Pathogenic	Lukas 2013^24^
p.Trp236Arg	Classic	1F	1F, 45–50	1 African- American	0	Pathogenic	Shabbeer 2006^25^
p.Cys202Ser		1F	1F, 45–50	1 East Asian	1F, 45–50	Likely pathogenic	Not reported
p.Ser201Ala		5: 4M, 1F	1F 55–60, 1M 55–60, 1M 60–65	5 East Asian	1F, 55–60	VUS	Not reported
p.Tyr200Cys		1F	Not known	1 Euro	0	Not reported	Benjamin 2017^26^
p.Ile198Thr	Later onset	2: 1M, 1F	1F, 60–65	2 Euro	1F, 60–65	Conflicting (P/LP/VUS)	Auray-Blais 2015^27^
p.Gly195Val	Later onset	1F	Not known	1 Euro	0	Not reported	Doi 2012^28^, ^29^
p.Asp153Asn		4: 1M, 3F	1F 50–55, 70–75; 1M 30–35	3 African American, 1 Other	1F, 75–80	VUS	Not reported
p.Gly144Asp		2F	1F <30	2African American	1F	Not reported	Li 2014^29^
p.Ala121Thr	Classic	1F	1F 50–55	1 Euro	0	VUS	Garman 2002^30^
p.Arg112His	Milder	2F	F 30–35	2 Euro	0	Path/Likely pathogenic	Eng 1994^31^
p.Asp83Asn	Variant	4F	F 40–45, 65–70	4 Euro	0	Conflicting, VUS/Likely Benign	Lukas 2013^24^
p.Thr41Ser		4–1M, 3F	M 50–55; F 40–45, 45–50, 75–80	4 Lat- American	3 (2F, 1M), 45–50, 50–55, 75–80	VUS	Not reported
p.Thr39Met		1F	Not known	1 Euro	1F	Not reported	Not reported
*N* = 20 different variants in 37 individuals		20 variants in 9M and 28F			11 variants in 2M, 12F		
		37/119,329			14/48,688		
		One in 3225			One in 3478		

Of the 20 variants found in gnomAD that fulfilled all the criteria for pathogenicity, 13 had previously been reported as disease-causing in the Fabry database (65%). Women had these variants more than 3 times as often as men (28:9 = 3.1). The median age for the males with a pathogenic variant was 50 years (range 30 to 75 years) and for the females was also 50 years (range <30 to 80 years).

Where Fabry disease had been reported previously, it was described as classical in 2 individuals (both women) and atypical with a milder onset in 4 (including men and women). These results were consistent with Fabry disease in the gnomAD cohort being found more often in women or individuals with milder or later onset disease.

Ancestry was European (non-Finnish) in 18, African American in 6, East Asian in 6, Latino in 4, and Other in 3. There were no individuals with a predicted pathogenic variant from the Ashkenazi Jewish, Finnish, or South Asian ancestries.

### Control Subset

The control data set comprised 73,032 alleles or 48,688 individuals. It included 11 predicted pathogenic variants from 14 people, which corresponded to a population frequency of 14 of 48,688 or 1 in 3478. These included 12 females and 2 males, with a much higher female-to-male ratio than in the overall cohort (6 compared with 3.1).

### Population Frequencies of Variants From Other Datasets

#### ClinVar

Seven variants in gnomAD were assessed in ClinVar as pathogenic or likely pathogenic (Supplementary Table S4). A further 6 were classified as Conflicting, where individual assessments were VUS and pathogenic or likely pathogenic. These were found in 27 individuals overall in gnomAD, which corresponded to an overall frequency of 27 of 119,329 or a population frequency of, at most, 1 in 4420.

#### Fabry Database

Forty-seven of the 100 (47%) variants found in gnomAD were also present in the Fabry database. Nineteen of these variants were labeled as a VUS or a possible VUS (?VUS) or were found in more than 5 individuals in gnomAD and hence were considered unlikely to be pathogenic and were excluded. Twenty-eight variants that were associated with classical or atypical Fabry disease, were not labeled ?VUS or a VUS, and occurred in ≤5 individuals were considered disease-causing in the Fabry database and were found in 45 individuals or in 1 in 2651 of the gnomAD cohort.

#### Fabry Expert Panel

Two variants found in gnomAD had been assessed by a Fabry expert panel who considered p.Arg363His to be pathogenic and associated with later onset disease. However, this variant did not fulfill our computational criteria for pathogenicity and was present in 10 apparently normal individuals. The other variant, p.Ala143Thr, was considered a VUS by the expert panel, did not fulfill our computational criteria and was found in 104 normal individuals. Neither variant was included in the calculations based on our assessment.

## Discussion

The population frequency of undiagnosed Fabry disease in gnomAD was 1 in 3225 based on a rigorous assessment of variant pathogenicity but without phenotypic data. The frequency in the gnomAD controls was 1 in 3478 suggesting minimal bias from the inclusion of patients with heart disease or stroke due to Fabry disease in the larger data set.

However, our population frequencies for Fabry disease from gnomAD are still underestimates because they do not include already-diagnosed individuals who had been excluded from gnomAD. The gnomAD cohort also did not indicate individuals with large rearrangements or deletions (although the constraint values for *GLA* suggest that these are uncommon), and noncanonical splice variants or the intronic changes that are detected less often with the commonly-used technique of whole exome sequencing. Importantly, analysis of our assessment criteria suggested that we underestimated the number of individuals with classical and especially later onset Fabry disease. Indeed, our criteria excluded the p.Arg363His variant, which was considered pathogenic by an expert panel.

Evaluation of our assessment strategy indicated that our population frequencies also underestimated the number of individuals with atypical disease or VUS that were actually pathogenic, and included most cases of classical disease.

Genomic variants in Fabry disease are difficult to assess for pathogenicity. Our approach was to use some commonly-accepted computational tools rather than to identify pathogenic variants at the standard required for clinical diagnosis. Reducing the testing stringency would have detected more variants associated with atypical disease and more normal variants; and that would have overestimated the population frequency. Diagnostic laboratories have the advantage of additional clinical data, family segregation, and possibly, biochemical studies to decide on pathogenicity. However, there are also reports of pathogenic variants where the classifications vary in different laboratories or have been revised subsequently by an expert panel.^32^ The disease may not be obvious in women, and some middle-aged men still have only mild features that are not recognizable as Fabry disease. Even where variants occur repeatedly in the Fabry database, it is not clear whether this is due to a founder effect or to a common normal variant. Missense variants found in individuals with reduced enzyme levels may still be normal.

The estimates of population frequencies calculated from pathogenic variants in the Fabry and ClinVar databases also have limitations. The Fabry database includes the variant evaluations made at submission over the past 20 years, and includes conflicting assessments. ClinVar includes many fewer pathogenic variants than the Fabry database; however, submissions are more recent and more likely to be accurate. Surprisingly, there was only about two-thirds overlap between pathogenic variants in these datasets.

Nevertheless, this is the first study that has demonstrated more pathogenic *GLA* variants in undiagnosed women than in men. With an X-linked disease, women are expected to have pathogenic variants twice as often as men, and the 3:1 ratio found here may have occurred because women were less likely to be diagnosed at recruitment into gnomAD, or excluded on the basis of known Mendelian disease or kidney failure. The proportion of women to men was even greater in the control cohort (6:1), indicating that the overrepresentation of women did not result from women recruited into gnomAD for cardiac disease or complications of undiagnosed Fabry disease. It may, however, have resulted from including fewer men with Fabry disease.

These results simply reflect that many women with Fabry disease are undiagnosed but still have the risks of kidney failure, heart disease, and stroke;^33^ and that this risk, population wise, is greater than for men.^2^ In addition, women pass on the disease-causing variant to half their sons and half their daughters.

The accuracy of our estimated population frequency depended on a rigorous assessment. Predicted pathogenic variants were presumed to be rare occurring in fewer than or equal to 5 people and affecting a coding region or splice site.

Overall, there was no difference in the population frequencies of Fabry disease in people of different ancestries except that predicted pathogenic variants were absent from people of South Asian, Ashkenazi Jewish, and Finnish backgrounds probably because of their small cohort size, and, in the case of Ashkenazim and Finns, their geographic and cultural isolation. We were not able to examine for the founder variant common in East Asian people because of its deep intronic nature.^34^

The strengths of this study were that the gnomAD cohort is relatively unbiased, the study included an assessment of the strategy for predicting pathogenicity, and that, for the first time, more women were recognized with a pathogenic Fabry variant than men. The limitations of this study were that our population frequency is still an underestimate because the whole exome sequencing used in gnomAD does not detect all variants, and our strategy was more sensitive for classical than atypical disease.

Genetic testing is the most sensitive method for the detection of *GLA* variants and therefore for the diagnosis of Fabry disease. Pathogenic variants that cause Fabry disease are commonly undiagnosed especially in women. Our criteria for pathogenicity may be useful in further assessment of variants from the Fabry database.

## Disclosure

All the authors declared no competing interests.

## Supplementary Material

Supplementary File (PDF)

**Table S1.** Assessment of *GLA* variants associated with (a) classical Fabry disease or (b) later onset atypical disease.

**Table S2.** Assessment of *GLA* variants classified as VUS in Fabry database.

**Table S3.** Assessment of *GLA* variants considered benign (benign/likely benign in ClinVar), common in gnomAD or benign in Fabry database.

**Table S4.** Assessment of all *GLA* variants in gnomAD using our strategy for pathogenicity.
