# Supplementary material for: Population Frequency of Undiagnosed Fabry Disease in the General Population
Source: Kidney Int Rep. 2023 Apr 17;8(7):1373–9. doi: 10.1016/j.ekir.2023.04.009 (PMC10334396; doi:10.1016/j.ekir.2023.04.009)
Supplement: Supplementary File (PDF) [file mmc1.pdf]

Supplementary Tables and Figures

Suppl Table 1: Assessment of *GLA* variants associated with (a) classical Fabry disease or (b)later onset atypical disease

Suppl Table 2: Assessment of *GLA* variants classified as VUS in Fabry db

Suppl Table 3: Assessment of *GLA* variants considered Benign (Benign/Likely Benign in Clin Var), common in gnomAD or Benign in Fabry db

Suppl Table 4: Assessment of all GLA variants in gnomAD using our strategy for pathogenicity

Suppl Table 1: Assessment of *GLA* variants associated with (a) classical Fabry disease or (b)later onset atypical disease

HGVS Consequence	Protein Consequence	Allele count	PP2 >0.80	SIFT 4G	Mutation Taster	Conserved	Conclusion	Reference
(a)c.103G>A	G35R	0	0.999	Damaging	Disease-causing	YES	Predicted pathogenic	Lukas 2013
c.126G>C	M42I	0	0.981	Damaging	Disease-causing	YES	Predicted pathogenic	Kobayashi 2019
c.145C>T	R49C	0	1	Damaging	Disease-causing	YES	Predicted pathogenic	Shin 2008
c.155G>A	C52Y	0	1	Damaging	Disease-causing	YES	Predicted pathogenic	Rigoldi (2014)
c.257A>G	Y86C	0	1	Damaging	Disease-causing	YES	Predicted pathogenic	Eng (1997)
c.317T>G	L106R	0	0.879	Damaging	Disease-causing	YES	Predicted pathogenic	Wattanasirichaigoon (2006)
c.394G>A	G132R	0	1	Damaging	Disease-causing	YES	Predicted pathogenic	Shabbeer (2002)
c.425G>A	C142Y	0	1	Damaging	Disease-causing	YES	Predicted pathogenic	Okumiya (1995)
c.431G>T	G144V	0	1	Damaging	Disease-causing	YES	Predicted pathogenic	Eng (1994)
c.465T>A	D155E	0	0.992	Damaging	Disease-causing	YES	Predicted pathogenic	Kobayashi (2019)
c.488G>T	G163V	0	1	Damaging	Disease-causing	YES	Predicted pathogenic	Garman (2002)
c.514T>C	C172R	0	1	Damaging	Disease-causing	YES	Predicted pathogenic	Saito (2013)
c.548G>C	G183A	0	0.995	Damaging	Disease-causing	YES	Predicted pathogenic	Filoni (2010)
c.747C>A	N249K	0	0.004	Tolerated	Polymorphism	NO	Predicted Benign	Duro (2014)
c.779G>C	G260A	0	1	Damaging	Disease-causing	YES	Predicted pathogenic	Garman (2002)
c.796G>A	D266N	0	1	Damaging	Disease-causing	YES	Predicted pathogenic	Lee (2010)
c.806T>C	V269A	0	1	Damaging	Disease-causing	No but similar	Predicted pathogenic	Davies (1993)
c.827G>C	S276T	0	0.93	Damaging	Disease causing	YES	Predicted pathogenic	Sawada(2020)
c.848A>C	Q283P	0	1	Damaging	Disease-causing	YES	Predicted pathogenic	Shabbeer (2006)
c.870G>A	M290I	1	0.983	Damaging	Disease-causing	YES	Predicted pathogenic	Lukas (2013)
c.890C>T	S297F	0	1	Damaging	Disease-causing	YES	Predicted pathogenic	Saito (2013)
c.928C>T	L310F	0	1	Damaging	Dsease-causing	No but similar	Predicted pathogenic	Lukas (2013)
c.947T>C	V316A	0	1	Tolerated	Disease-causing	No but similar	Predicted Benign	Lukas (2016)
c.982G>A	G328R	0	1	Damaging	Disease-causing	YES	Predicted pathogenic	Saito (2013)
c.1016T>A	V339E	0	0.999	Damaging	Disease-causing	YES	Predicted pathogenic	Kobayashi (2019)
c.1025G>A	R342Q	0	1	Damaging	Disease-causing	YES	Predicted pathogenic	Pasqualim (2014)
c.1066C>T	R356W	0	0.999	Damaging	Disease-causing	NO	Predicted Benign	Pasqualim (2014)
c.1081G>C	G361R	0	1	Tolerated	Disease-causing	NO	Predicted Benign	Garman (2002)

c.361G>C	A121P	0	0.998	Damaging	Dsease-causing	YES	Predicted pathogenic	Kotanko (2004)
c.1078G>A	G360S	0	1	Damaging	Disease-causing	NO	Predicted Benign	Dobrovolny (2005)
(b) c.335G>A	R112H	2	1	Damaging	Disease-causing	YES	Predicted pathogenic	Shimotori (2008)
c.58G>C	A20P	0	0.877	Tolerated	Disease-causing	No but similar	Predicted Benign	Nakao 1995
c.194G>C	S65T	0	0.957	Damaging	Disease-causing	YES	Predicted pathogenic	Garman (2002)
c.214A>G	M72V	0	0.755	Tolerated	Disease-causing	YES	Predicted Benign	Saito (2013)
c.290C>T	A97V	0	0.147	Tolerated	Disease-causing	YES	Predicted Benign	Saito (2013)
c.613C>T	P205S	0	1	Damaging	Disese-causing	YES	Predicted pathogenic	Pan (2016)
c.629C>T	P210L	0	0.975	Tolerated	Disease-causing	NO	Predicted Benign	Saito (2013)
c.676T>G	W226G	0	1	Damaging	Disease-causing	YES	Predicted pathogenic	Choi (2017)
c.902G>A	R301Q	0	1	Damaging	Benign	YES	Predicted Benign	Germain (2002)
c.924A>C	K308N	0	1	Damaging	Benign	YES	Predicted Benign	Koulousios (2017)
c.890C>G	M296I	0	0.084	Damaging	Disease-causing	YES	Predicted Benign	Garman (2002)
c.868A>C	M290V	0	1	Tolerated	Disease-causing	YES	Predicted Benign	Pan 92016)
c.835C>G	Q279E	0	0.993	Damaging	Disease-causing	YES	Predicted pathogenic	Okumiya (1995)
c.819T>A	F273L	0	1	Damaging	Disease-causing	YES	Predicted pathogenic	Zhang (2007)
c.717A>G	I239M	0	0.999	Damaging	Disease-causing	No but similar	Predicted pathogenic	Csanyi (2017)
c.713G>A	S238N	0	1	Tolerated	Disease-causing	NO	Predicted Benign	Monserrat (2007)
c.682A>C	N228H	0	1	Damaging	Disease-causing	YES	Predicted pathogenic	Vanga 92020)
c.676T>G	W226G	0	1	Damaging	Disease-causing	YES	Predicted pathogenic	Choi (2017)
c.657C>G	I219M	0	0.998	Damaging	Disease-causing	No but similar	Predicted pathogenic	Nampoothiri (2020)
c.644A>G	N215S	1	0.291	Damaging	Benign	YES	Predicted Benign	Eng 91994)
c.623T>G	M208R	0	0.128	Tolerated	Benign	NO	Predicted Benign	Sakaruba (2018)
c.587G>C	R196T	0	1	Damaging	Disease causing	YES	Predicted pathogenic	Pan (2016)
c.584G>T	G195V	1	1	Damaging	Disease causing	YES	Predicted pathogenic	Doi (2012)
c.520T>G	C174G	0	0.581	Tolerated	Benign	YES	Predicted Benign	Serebrinsky (2015)
c.511G>A	G171S	0	1	Damaging	Disease-causing	YES	Predicted pathogenic	Koulousios (2017)
c.479C>A	A160D	0	1	Damaging	Disese -causing	YES	Predicted pathogenic	Pan (2016)
c.454T>C	Y152H	0	1	Tolerated	Disease causing	NO	Predicted Benign	Battaglia (2019)
c.371T>G	V124G	0	1	Damaging	Disease causing	YES	Predicted pathogenic	Pan (2016)

c.337T>C	F113L	0	1	Damaging	Disease causing	YES	Predicted pathogenic	Saito 92013)
c.335G>T	R112L	0	1	Damaging	Disease causing	YES	Predicted pathogenic	Degirmenci (2017)
c.288G>A	M96I	0	0.002	Tolerated	Benign	NO	Predicted Benign	Sakuraba (2018)
c.263A>G	Y88C	0	1	Damaging	Disease causing	YES	Predicted pathogenic	Pan (2016)
c.218C>T	A73V	0	0.722	Damaging	Diseae-causing	YES	Predicted Benign	Lukas 92013)
c.207C>A	F69L	0	0.999	Damaging	Disease-causing	YES	Predicted pathogenic	Umeda (20150
c.154T>C	C52R	0	1	Damaging	Disease-causing	YES	Predicted pathogenic	Germain (1996)
c.153G>A	M51I	0	0.067	Damaging	Benign	NO	Predicted Benign	Cammarata (2015)
c.107T>C	L36S	0	1	Damaging	Disease causing	YES	Predicted pathogenic	Erdos 92008)

Suppl Table 2: Assessment of *GLA* variants classified as VUS in Fabry db

HGVS consequence	Protein consequence	Allele frequency	PP2≥ 0.95	SIFT 4G	Mutation Taster	Conserved	Conclusion	Reference
c.902G>A	R301Q	0	1	Damaging	Disease-causing	YES	Predicted pathogenic	Lee (2010)
c.685T>G	F229V	0	0.981	Damaging	Disease-causing	NO but similar	Predicted pathogenic	Sawada (2020)
c.714T>A	S238R	0	1	Damaging	Disease-causing	YES	Predicted pathogenic	Sawada(2020)
c.596T>C	V199A	3	0.998	Tolerated	Disease-causing	YES	Predicted Benign	Pan (2016)
c.1153A>G	T385A	48	0.861	Tolerated	Disease-causing	NO	Predicted Benign	Duro (2018)
c.1244T>C	L415P	0	1	Damaging	Disease causing	NO	Predicted Benign	Rozenfeld (2006)
c.1250T>C	L417P	0	1	Damaging	Polymorphism	YES	Predicted Benign	Sakuraba (2018)
c.1067G>A	R356Q	2	0.376	Tolerated	Polymorphism	NO	Predicted Benign	Liao (2018)
c.1084C>A	P362T	0	0.983	Damaging	Disease-causing	YES	Predicted pathogenic	Liao (2018)
c.1102G>A	A368T	9	0	Tolerated	Polymorphism	NO	Predicted Benign	Varela (2020)
c.1171A>G	K391E	0	0.859	Tolerated	Polymorphism	NO	Predicted Benign	Sawada (2020)
c.1172A>C	K391T	0	0.754	Tolerated	Polymorphism	NO	Predicted Benign	Liao (2018)
c.1231G>A	G411S	0	1	Damaging	Disease-causing	YES	Predicted pathogenic	Sawada (2020)
c.911G>C	S304T	0	0.632	Tolerated	Polymorphism	YES	Predicted Benign	Liao (2018)
c.659G>A	R220Q	0	0.891	Tolerated	Disease-causing	NO	Predicted Benign	Varela (20200
c.683A>G	N228S	0	1	Damaging	Disease-causing	YES	Predicted pathogenic	Varela (20201
c.685T>G	F229V	0	0.981	Damaging	Disease-causing	No but similar	Predicted pathogenic	Sawada (2020)
c.714T>A	S238R	0	1	Damaging	Disease-causing	YES	Predicted pathogenic	Sawada (2020)
c.725T>C	I242T	0	0.6	Damaging	Polymorphism	YES	Predicted Benign	Sawada (2020)
c.596T>C	V199A	3	0.998	Tolerated	Disease-causing	YES	Predicted Benign	Pan (2016)
c.605G>T	C202F	0	1	Damaging	Disease-causing	YES	Predicted pathogenic	Sawada (2020)
c.625T>C	W209R	0	0.016	Tolerated	Polymorphism	NO	Predicted Benign	Sawada (2020)
c.376A>G	S126G	74	0.277	Tolerated	Disease-causing	NO but similar	Predicted Benign	Duro 92018)
c.428C>T	A143V	0	0.999	Damaging	Disease causing	NO	Predicted Benign	Sawada (2020)
c.493G>A	D165N	0	1	Damaging	Disease-causing	YES	Predicted pathogenic	Sawada (2020)
c.538T>G	L180V	0	0.346	Damaging	Disease-causing	NO	Predicted Benign	Sawada (2020)
c.301G>A	D101N	0	0.004	Tolerated	Disease causing	YES	Predicted Benign	Lin (2018)

c.316C>T	L106F	0	0.017	Damaging	Disease causing	YES	Predicted Benign	van der Tol (2015)
c.322G>A	A108T	0	0.981	Damaging	Disease causing	YES	Predicted pathogenic	Liao (2018)
c.353G>A	R118H	3	0.027	Tolerated	Polymorphism	NO but similar	Predicted Benign	Duro (2018)
c.13A>G	N5D	1	0.023	Tolerated	Polymorphism	NO	Predicted Benign	Duro (2018)
c.164A>G	D55G	0	0.95	Damaging	Disease-causing	NO but similar	Predicted pathogenic	Frabasil (2019)
c.178C>T	P60S	0	1	Damaging	Disease-causing	YES	Predicted Benign	Laio (2018)
c.179C>T	P60L	0	0.979	Damaging	Disease-causing	YES	Predicted Pathogenic	Smid 92015)
c.43G>A	A15T	1	0.309	Tolerated	Polymorphism	YES	Predicted Benign	Ebrahim (2012)
c.70T>A	W24R	0	0.347	Tolerated	Polymorphism	NO	Predicted Benign	Al-Thihli (2012)

Suppl Table 3: Assessment of *GLA* variants considered Benign (Benign/Likely Benign in Clin Var), common in gnomAD or Benign in Fabry db

HGVS consequence	Protein consequence	Allele frequency	PP2 > 0.95	SIFT	Mutation Taster	Conserved	Conclusion	Reference
c.1153A>G	T385A	103	0.861	Tolerated	Disease-causing	NO	Benign	
c.1060A>G	I354Val	2	0	Tolerated	Benign	NO	Benign	
c.721A>G	S241G	0	0.088	Tolerated	Benign	NO	Benign	
c.416A>G	N139S	34	0.013	Tolerated	Polymorphism	NO	Benign	
c.320A>G	Q107R	0	0.005	Tolerated	Benign	NO	Benign	
c.937G>T	D313Y	624	0.996	Damaging	Disease-causing	NO	Benign	
c.865A>G	I289V	33	0.994	Tolerated	Disease-causing	YES	Benign	
c.427G>A	A143T	103	0.999	Damaging	Disease-causing	NO	Benign	Smid (2015)
c.416A>G	N139S	34	0.013	Tolerated	Polymorphism	NO	Benign	
c.376A>G	S126G	74	0.277	Tolerated	Disease-causing	No but similar	Benign	
c.352C>T	R118C	48	1	Damaging	Polymorphism	No but similar	Benign	
c.8T>C	L3P	62	0.003	Damaging	Polymorphism	No but similar	Benign	
c.196G>C	E66Q	22	0.939	Damaging	Disease-causing	YES	Benign	
c.1102G>A	A368T	19	0	Tolerated	Polymorphism	NO	Benign	
c.1196G>C	W399S	15	0.979	Tolerated	Polymorphism	NO	Benign	
c.1088G>A	R363H	10	0.406	Tolerated	Polymorphism	NO	Benign	
c.196G>C	E66Q	22	0.939	Uncertain	Uncertain	YES	Benign	Ihii (2007)
c.214A>G	M72V	0	0.755	Benign	Uncertain	YES	Benign	Takata (1997)
c.352C>T	R118C	48	1	Damaging	Polymorphism	NO	Benign	Ferreira (2015)
c.937G>T	D313Y	624	0.996	Damaging	Disease-causing	NO	Benign	Niemann (2013)

Suppl Table 4: Assessment of all GLA variants in gnomAD using our strategy, Clinvar and the Fabry database to assess pathogenicity

Transcript Consequence	Protein Consequence	CADD	PP2 >0.95	SIFT 4G <0.05	Mutation Taster	Conserved	ClinVar Clinical Significance	Predicted Conclusion	Allele Count	Controls	Reference from Fabry db
c.1285C>T	p.Leu429Phe	23.6	0.001	Damaging	Polymorphism	NO		Benign	1	0	Not reported
c.1280A>T	p.Asp427Val	5.1	0.002	Tolerated	Polymorphism	NO		Benign	1	1	Not reported
c.1279G>A	p.Asp427Asn	11.17	0	Tolerated	Polymorphism	NO		Benign	1	1	Not reported
c.1261A>G	p.Met421Val	5.2	0.036	Tolerated	Polymorphism	NO	Conflicting/Benign	Benign	4	1	Missense, Benjamin 2017, x1
c.1237G>A	p.Val413Ile	22.2	0.01	Tolerated	Disease-causing	YES		Benign	1		Not reported
c.1196G>C	p.Trp399Ser	17.13	0.979	Tolerated	Polymorphism	NO	VUS	Benign	15	8	Missense, Lukas 2016, x1
c.1192G>A	p.Glu398Lys	10.5	0.032	Tolerated	Polymorphism	No (but : similar)	VUS	Benign	2		Missense, Shabbeer 2002, x1
c.1184G>C	p.Gly395Ala	24.9	0.999	Damaging	Disease-causing	YES	VUS	Predicted pathogenic	2	2	Missense, Bono 2011, x2
c.1175G>C	p.Arg392Thr	8.602	0.008	Tolerated	Polymorphism	NO	VUS	Benign	5	1	Not reported
c.1168G>A	p.Val390Met	10.33	0.883	Tolerated	Polymorphism	NO		Benign	1		Missense, Lukas 2013
c.1159C>T	p.Leu387Phe	12.08	0.946	Tolerated	Disease-causing	NO (but : similar)		Benign	1		Not reported
c.1154C>T	p.Thr385Ile	19.86	0.989	Tolerated	Disease-causing	NO	VUS	Benign	1	1	Not reported
c.1153A>G	p.Thr385Ala		0.861	Tolerated	Disease-causing	NO	Benign/LB	Benign	103	48	Lukas 2013, ? VUS, x2
c.1112C>G	p.Ser371Cys	18.08	0.947	Damaging	Disease-causing	NO (but . similar)		Benign	1	0	Not reported
c.1102G>C	p.Ala368Pro	0	0.001	Tolerated	Polymorphism	NO	Confl/ probably Benign	Benign	4	2	Not reported
c.1102G>A	p.Ala368Thr		0	Tolerated	Polymorphism	NO	Confl/Benign	Benign	19	9	?? VUS, Lukas 2013, x 3
c.1093T>A	p.Tyr365Asn		0.99	Tolerated	Polymorphism	YES	VUS	Benign	2		Not reported
c.1091C>T	p.Ser364Phe		0.001	Tolerated	Polymorphism	NO		Benign	1		Not reported
c.1088G>A	p.Arg363His	8.38	0.406	Tolerated	Polymorphism	NO	Confl/Pathogenic	Benign	10	4	Missense, Cooper 2002, x8
c.1087C>T	p.Arg363Cys		0.981	Tolerated	Polymorphism	NO	Pathogenic/Likely pathogenic	Benign	2	2	Pathogenic, Shabbeer 2002, x2, Classical, Germain
c.1079G>C	p.Gly360Ala	25.1	1	Damaging	Disease-causing	NO		Benign	1	0	Not reported

c.1078G>T	p.Gly360Cys	25.5	1	Damaging	Disease-causing	NO		Benign	1	1	Pathogenic, Missense, Lin 2008, x8
c.1067G>A	p.Arg356Gln		0.376	Tolerated	Polymorphism	NO	Confl/pathogenic	Benign	2		Missense, HWU, 2009, ? VUS, x 4
c.1060A>G	p.Ile354Val		0	Tolerated	Polymorphism	NO	Likely benign	Benign	2	2	Not reported
c.1057A>G	p.Met353Val		0.053	Tolerated	Polymorphism	NO (but similar)	VUS	Benign	1	1	Not reported
c.1055C>G	p.Ala352Gly	16.28	0.995	Tolerated	Disease-causing	NO (but . similar)	VUS	Benign	1	1	Missense, Lukas 2016 x1
c.1028C>T	p.Pro343Leu	24.1	0.999	Damaging	Disease-causing	YES	Confl/pathogenic	Predicted Pathogenic	1	1	Missense, Mills 2005 x 1
c.1027C>T	p.Pro343Ser	25.6	1	Damaging	Disease-causing	YES		Predicted Pathogenic	1	0	Not reported
c.1001G>A	p.Gly334Glu		0	Tolerated	Polymorphism	NO		Benign	4	3	Missense, Scott 2013 x1
c.995G>A	p.Arg332Lys		0.06	Tolerated	Polymorphism	NO (but : similar)		Benign	5		Not reported
c.994A>G	p.Arg332Gly		0.084	Tolerated	Polymorphism	NO (but : similar)		Benign	5	1	Not reported
c.991C>T	p.Leu331Phe		0.036	Tolerated	Polymorphism	NO (but : similar)	Confl/Benign	Benign	3	2	Not reported
c.946G>A	p.Val316Ile		0.33	Tolerated	Disease-causing	NO (but : similar)	VUS	Benign	1	1	Missense, Lukas 2013
c.946G>C	p.Val316Leu		0.393	Tolerated	Disease-causing	NO (but : similar)		Benign	1	1	Not reported
c.937G>A	p.Asp313Asn		0.001	Tolerated	Polymorphism	NO	Confl/Benign	Benign	1		Lukas 2016, x1
c.937G>T	p.Asp313Tyr		0.996	Damaging	Disease-causing	NO	Confl/Benign	Benign	624	254	Eng 1993, ? Benign, x 27
c.928C>G	p.Leu310Val		0.988	Tolerated	Disease-causing	NO (but :similar)		Benign	1		later onset Choi, 2017
c.870G>C	p.Met290Ile	26.9	0.983	Damaging	Disease-causing	YES	Confl/pathogenic	Predicted Pathogenic	1	1	Not reported, but Classic disease at this site
c.868A>C	p.Met290Leu		0.924	Tolerated	Disease-causing	YES	Confl/pathogenic	Benign	2	1	Andreotti, 2011; female x3
c.865A>G	p.Ile289Val		0.994	Tolerated	Disease-causing	YES	Confl/Benign	Benign	33	12	Lukas 2016, female x1
c.853G>A	p.Ala285Thr	29.3	1	Damaging	Disease-causing	YES		Predicted Pathogenic	1	0	Not reported

c.790G>A	p.Asp264Asn	32	1	Damaging	Disease-causing	YES	VUS	Predicted Pathogenic	1	1	Lukas 2013, x1
c.755G>C	p.Arg252Thr		0.001	Tolerated	Polymorphism	NO	Conf/Benign	Benign	4		Not reported
c.745A>T	p.Asn249Tyr		0.004	Tolerated	Polymorphism	NO		Benign	1	1	Not reported
c.736A>T	p.Thr246Ser		0.501	Tolerated	Disease-causing	NO		Benign	1	1	Not reported
c.719delA	p.Lys240ArgfsTer29						Pathogenic	Predicted Pathogenic	1	1	Lukas 2013, x 1
c.715A>T	p.Ile239Leu		0.086	Tolerated	Disease-causing	NO (but :similar)		Benign	1		Not reported
c.706T>C	p.Trp236Arg	28.2	1	Damaging	Disease-causing	YES	Pathogenic	Predicted Pathogenic	1	0	Classic, Shabbeer 2006
c.644A>G	p.Asn215Ser		0.291	Damaging	Disease-causing	YES	Pathogenic	Benign	1		? Mild to severe, Eng 1993, x 22
c.635A>G	p.Gln212Arg		0.001	Tolerated	Polymorphism	YES	VUS	Benign	6	4	Not reported
c.631T>C	p.Phe211Leu		0.009	Tolerated	Polymorphism	NO		Benign	1		Not reported
c.619T>C	p.Tyr207His	25.4	1	Damaging	Disease-causing	YES		Benign	6	3	Missense Benjamin 2017
c.605G>C	p.Cys202Ser	25	1	Damaging	Disease-causing	YES	Likely pathogenic	Predicted pathogenic	1	1	Not reported
c.601T>G	p.Ser201Ala	28.2	0.996	Damaging	Disease-causing	YES	VUS	Predicted pathogenic	5	1	Not reported
c.599A>G	p.Tyr200Cys		1	Damaging	Disease-causing	YES		Predicted Pathogenic	1	0	Missense Benjamin 2017
c.596T>C	p.Val199Ala		0.998	Tolerated	Disease-causing	YES	VUS	Benign	3	2	? VUS Pan 2016
c.593T>C	p.Ile198Thr	27.2	1	Damaging	Disease-causing	YES	Confl/Pathogenic	Pathogenic in Germain	2	1	Later onset, Auray-Blais 2015, Germain
c.590G>A	p.Ser197Asn		0.899	Damaging	Disease-causing	NO (but.similar)	VUS	Benign	1		Not reported
c.584G>T	p.Gly195Val	24.7	1	Damaging	Disease-causing	YES		Pathogenic	1	0	Later onet Doi, 2012, x 2
c.579G>C	p.Arg193Ser		0.967	Tolerated	Polymorphism	NO	VUS	Benign	3	1	Not reported
c.566T>G	p.Leu189Trp		0.979	Damaging	Polymorphism	NO	VUS	Benign	2	1	Not reported
c.544G>A	p.Asp182Asn		0	Tolerated	Disease-causing	NO (but : similar)	VUS	Benign	1		Not reported
c.525C>G	p.Asp175Glu		0.058	Tolerated	Polymorphism	NO (but .similar)	Confl/Benign	Benign	6		Lukas 2013, missense x1

c.525C>A	p.Asp175Glu		0.058	Tolerated	Polymorphism	NO (but.similar)	VUS	Benign	1		Not reported
c.457G>A	p.Asp153Asn	25.2	0.991	Damaging	Disease-causing	NO (but: similar)	VUS	Predicted Pathogenic	4	1	Not reported
c.431G>A	p.Gly144Asp	27.6	0.991	Damaging	Disease-causing	YES		Predicted Pathogenic	2	1	Li, 2014; in female, x 1
c.427G>A	p.Ala143Thr		1	Disease-causing	Damaging	NO (but.similar)	Confl/pathogenic/Benign	Benign	104	55	Eng 1997; x 21 probably benign (Germain)
c.419A>C	p.Lys140Thr		0.137	Tolerated	Polymorphism	NO	VUS	Benign	5	1	Not reported
c.416A>G	p.Asn139Ser		0.013	Tolerated	Polymorphism	NO	Likely benign	Benign	34	16	Havndrup 2010, missense x2
c.377G>T	p.Ser126Ile		0.997	Damaging	Disease-causing	NO		Predicted Pathogenic	1		Missense Lukas 2013 x1
c.376A>G	p.Ser126Gly		0.277	Tolerated	Disease-causing	NO	Confl/Benign	Benign	74		? VUS, x8, Altarescu 2001
c.365A>G	p.Asn122Ser		0.003	Damaging	Disease-causing	NO		Benign	1		Not reported
c.361G>A	p.Ala121Thr	25.2	0.99	Damaging	Disease-causing	YES	VUS	Predicted Pathogenic	1	0	Garman 2002, ? Severe, x2
c.353G>A	p.Arg118His		0.027	Tolerated	Polymorphism	NO (but : similar)	VUS	Benign	3		? Missense, ?VUS Lukas 2013
c.352C>T	p.Arg118Cys		1	Damaging	Polymorphism	NO (but : similar)	Confl/Path/Benign	Benign	48		? later onset Spada 2006, x7
c.341C>T	p.Pro114Leu		0.88	Damaging	Disease-causing	YES	VUS	Benign	1		Not reported
c.335G>A	p.Arg112His	30	1	Damaging	Disease-causing	YES	Pathogenic/Likely pathogenic	Predicted pathogenic	2	0	Later onset, Milder variants, x 10, Eng 1994
c.313A>G	p.Arg105Gly		0.051	Damaging	Disease-causing	YES	VUS	Benign	1		Not reported
c.276T>A	p.Asp92Glu		0.786	Damaging	Disease-causing	YES	Pathogenic	Benign	1		Not reported
c.247G>A	p.Asp83Asn	25.8	0.968	Damaging	Disease-causing	NO (but :similar)	Confl/Benign	Predicted Pathogenic	4	0	Missense x 3, Lukas 2013
c.212A>G	p.Glu71Gly		0.711	Tolerated	Disease-causing	NO (but: similar)	VUS	Benign	1	1	Missense, Lukas 2016
c.208A>G	p.Met70Val		0.18	Tolerated	Disease-causing	YES	VUS	Benign	1		Not reported
c.196G>C	p.Glu66Gln		0.939	Damaging	Disease-causing	YES	Confl/Benign	Benign	22	12	? Not pathogenic, Kobayashi 2012
c.122C>G	p.Thr41Ser	27.7	0.964	Damaging	Disease-causing	YES	VUS	Predicted Pathogenic	4	3	Not reported

c.116C>T	p.Thr39Met	22.9	1	Damaging	Disease-causing	YES		Predicted pathogenic	1	1	Not reported
c.89G>C	p.Arg30Thr		0.001	Tolerated	Polymorphism	YES		Benign	2	1	Not reported
c.85G>A	p.Ala29Thr		0.007	Tolerated	Polymorphism	NO (but. similar)	VUS	Benign	2		Not reported
c.73G>A	p.Asp25Asn		0.048	Tolerated	Polymorphism	NO	VUS	Benign	1		Not reported
c.61C>T	p.Leu21Phe		0.062	Tolerated	Polymorphism	YES	Likely Benign	Benign	2	2	Misense, Heo 2009
c.44C>T	p.Ala15Val		0.033	Tolerated	Polymorphism	YES		Benign	1	1	Not reported but alternative substitution (Ala15Glu) causes Classic disease
c.43G>A	p.Ala15Thr		0.309	Tolerated	Polymorphism	YES	VUS	Benign	1		? VUS, Ebrahim 2012, Ala15Glu causes Classic disease
c.32G>C	p.Gly11Ala		0.003	Tolerated	Polymorphism	NO	VUS	Benign	3	2	Not reported
c.32G>A	p.Gly11Asp		0.745	Tolerated	Polymorphism	NO		Benign	1	1	Not reported
c.31G>A	p.Gly11Ser		0.025	Tolerated	Polymorphism	NO		Benign	1	1	Not reported
c.31G>C	p.Gly11Arg		0.745	Tolerated	Polymorphism	NO		Benign	1	1	Not reported
c.26A>G	p.His9Arg		0	Tolerated	Polymorphism	NO	Confl/Benign	Benign	1	1	Not reported
c.21A>C	p.Glu7Asp		0	Tolerated	Polymorphism	NO	VUS	Benign	1		Not reported
c.13A>G	p.Asn5Asp		0.023	Tolerated	Polymorphism	NO	VUS	Benign	1	1	?VUS, Duro, 2018
c.8T>C	p.Leu3Pro		0.003	Damaging	Polymorphism	NO	Confl/Benign	Benign	62	23	Lukas 2013
c.5A>C	p.Gln2Pro		0	Tolerated	Polymorphism	NO		Benign	1		Not reported

Figure 1: Distribution of variants in GLA throughout the protein

Likely pathogenic and pathogenic variants in *GLA* from Clin Var demonstrating that variants are found throughout the gene. Above the horizontal are variants from Clin Var and below the horisonal are those also found in gnomAD

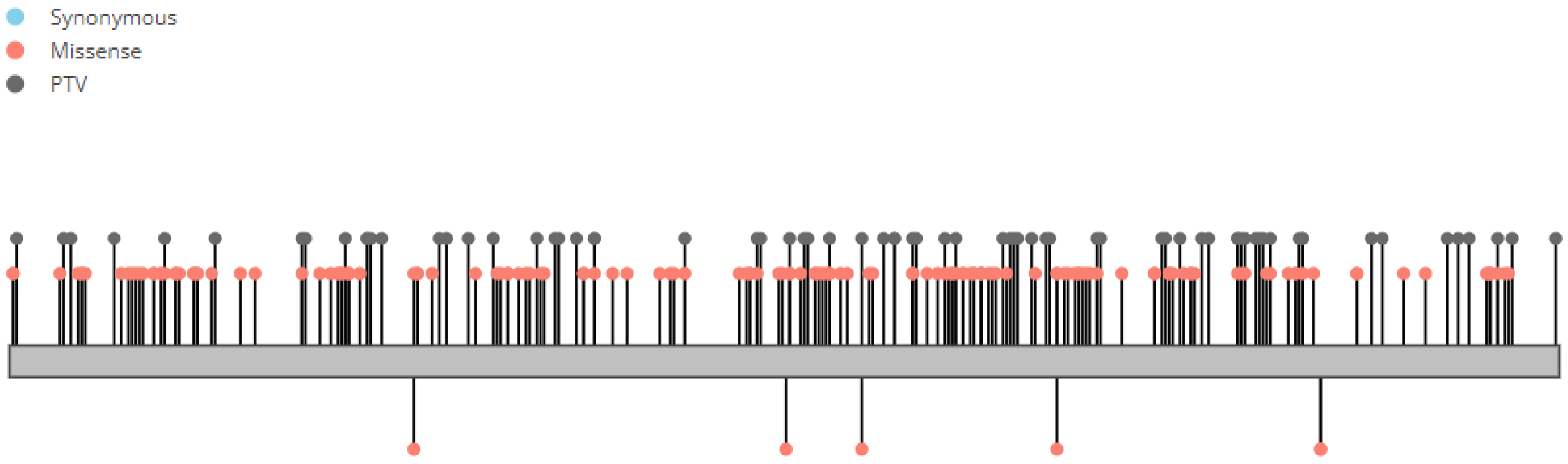

From https://simple-clinvar.broadinstitute.org/

Figure 2: *GLA* Clustal for Fabry disease with alignment of human mouse and chicken protein sequences

```
Hs      --MQLRNPELHLGCALALR-----------FLALVSWDIPGARALDNGLARTPTMGWLHW    47
Mm      MAMKLLSRDTRLVCELALC-----------PLALVFWSILGVRALDNGLARTPTMGWLHW    49
Gg      MRCHVSGPGRGEGCRRALVEAAAGMGVPAAVAVLAAALALPARGLENGLARTPPMGWLAW    60
           :: .      *  **              .*.      .*.*:******* **** *

Hs      ERFMCNLDCQEEPDSCISEKLFMEMAELMVSEGWKDAGYEYLCIDDCWMAPQRDSEGRLQ    107
Mm      ERFMCNLDCQEEPDACISEQLFMQMAELMVSDGWRDAGYDYLCIDDCWMAPERDSKGRLQ    109
Gg      ERFRCNVNCREDPRQCISEMLFMEMADRIAEDGWRELGYKYINIDDCWAAKQRDAEGRLV    120
        *** **::*:*:*  **** ***:**: :..:**:: **.*: ***** * :**::***

Hs      ADPQRFPHGIRQLANYVHSKGLKLGIYADVGNKTCAGFPGS-FGYYDIDAQTFADWGVDL    166
Mm      ADPQRFPSGIKHLANYVHSKGLKLGIYADVGNKTCAGFPGS-FGSYDIDAQTFADWGVDL    168
Gg      PDPERFPRGIKALADYVHARGLKLGIYGDLGRLTCGGYPGTTLDRVEQDAQTFAEWGVDM    180
         **:*** **: **:***::*******.*:*. **.*:**: :.  : ******:****:

Hs      LKFDGCYCDSLENLADGYKHMSLALNRTGRSIVYSCEWPLYMWPF-QKPNYTEIRQYCNH    225
Mm      LKFDGCHCDSVVSLENGYKYMALALNRTGRSIVYSCEWPLYLRPF-HKPNYTDIQYYCNH    227
Gg      LKLDGCYSSGK-EQAQGYPQMARALNATGRPIVYSCSWPAYQGGLPPKVNYTLLGEICNL    239
        **:***:...  .  :**  *: *** *** *****.** *   :  * *** :   **

Hs      WRNFADIDDSWKSIKSILDWTSFNQERIVDVAGPGGWNDPDMLVIGNFGLSWNQQVTQMA    285
Mm      WRNFDDVYDSWESIKNILSWTVVYQKEIVEVAGPGSWNDPDMLVIGNFGLSWDQQVTQMA    287
Gg      WRNYDDIQDSWDSVLSIVDWFFTNQDVLQPFAGPGHWNDPDMLIIGNFGLSYEQSRSQMA    299
        ***: *: ***.*: .*:.*    *. :  .**** *******:*******::*. :***

Hs      LWAIMAAPLFMSNDLRHISPQAKALLQDKDVIAINQDPLGKQGYQL-RQGDNFEVWERPL    344
Mm      LWAIMAAPLLMSNDLRQISSQAKALLQNKDVIAINQDPLGKQGYCF-RKENHIEVWERPL    346
Gg      LWTIMAAPLLMSTDLRTISPSAKKILQNRLMIQINQDPLGIQGRRIIKEGSHIEVFLRPL    359
        **:******:**.*** ** .** :**:: :* ******* **  : :: .::**: ***

Hs      SGLAWAVAMINRQEIGGPRSYTIAVASLGKGVACNPACFITQLLPVKRKLGFYEWTSRLR    404
Mm      SNLAWAVAVRNLQEIGGPCPYTIQISSLGRGLACNPGCIITQLLPEKVHLGFYEWTLTLK    406
Gg      SQAASALVFFSRR-TDMPFRYTTSLAKLGFPMGAA--YEVQDVYSGKIISGL-KTGDNFT    415
        *  * *:.. . :  . *  **  ::.**  :..     : ::   *   *: :    :

Hs      SHINPTGTVLLQLENTMQMSLKD---------LL      429
Mm      TRVNPSGTVLFRLER-------------------      421
Gg      VIINPSGVVMWYLCPKALLIQQQAPGGPSRLPLL      449
          :**:*.*:  *
```

Amino acids are coloured as follows: Red – small and hydrophobic; Blue – acidic; Magenta – basic; Green – hydroxyl plus sulfhydryl plus amine plus Gly; Grey – unusual amino or imino acids.

Alignment is indicated by symbols under aligned residues: Asterix – single fully conserved residue; : colon – conservation between groups with strongly similar properties; . period – conservation between groups of weakly similar properties
